# Bioelectricity Generation and Bioremediation of an Azo-Dye in a Microbial Fuel Cell Coupled Activated Sludge Process

**DOI:** 10.1371/journal.pone.0138448

**Published:** 2015-10-23

**Authors:** Mohammad Danish Khan, Huda Abdulateif, Iqbal M. Ismail, Suhail Sabir, Mohammad Zain Khan

**Affiliations:** 1 Environmental Research Laboratory, Department of Chemistry, Aligarh Muslim University, Aligarh 202 002, India; 2 Centre for Excellence in Environmental Studies, King Abdul Aziz University, Jeddah, Kingdom of Saudi Arabia; National Environmental Engineering Research Institute CSIR, INDIA

## Abstract

Simultaneous bioelectricity generation and dye degradation was achieved in the present study by using a combined anaerobic-aerobic process. The anaerobic system was a typical single chambered microbial fuel cell (SMFC) which utilizes acid navy blue r (ANB) dye along with glucose as growth substrate to generate electricity. Four different concentrations of ANB (50, 100, 200 and 400 ppm) were tested in the SMFC and the degradation products were further treated in an activated sludge post treatment process. The dye decolorization followed pseudo first order kinetics while the negative values of the thermodynamic parameter ∆G (change in Gibbs free energy) shows that the reaction proceeds with a net decrease in the free energy of the system. The coulombic efficiency (CE) and power density (PD) attained peak values at 10.36% and 2,236 mW/m^2^ respectively for 200 ppm of ANB. A further increase in ANB concentrations results in lowering of cell potential (and PD) values owing to microbial inhibition at higher concentrations of toxic substrates. Cyclic voltammetry studies revealed a perfect redox reaction was taking place in the SMFC. The pH, temperature and conductivity remain 7.5–8.0, 27(±2°C and 10.6–18.2 mS/cm throughout the operation. The biodegradation pathway was studied by the gas chromatography coupled with mass spectroscopy technique, suggested the preferential cleavage of the azo bond as the initial step resulting in to aromatic amines. Thus, a combined anaerobic-aerobic process using SMFC coupled with activated sludge process can be a viable option for effective degradation of complex dye substrates along with energy (bioelectricity) recovery.

## Introduction

Microbial fuel cells (MFCs) are the bioelectrochemical systems (BES) that harness the energy stored in chemical bonds in to electrical energy through catalytic action of microorganisms. The microbial conversion of organic substrate such as higher organics to acetate produces electrons which are transferred to anode [1]. These electrons then flow towards cathode linked by a conductive material containing a resistor [2,3]. Electrons are transferred to the anode by means of electron mediators or shuttles such as ABTS 2,2'-azino-bis(3-ethylbenzothiazoline-6-sulphonic acid) [4],by direct membrane associated electron transfer[5]or through nanowires produced by bacteria [6].In addition to contaminant degradation, the system offers electricity generation and reduction of metal ions in the cathodic chambers e.g. Mn (IV) to Mn (II) [1].The microorganisms consume a part of energy for growth while utilizes the rest for generating electricity, therefore the sludge production is quite, which is an added advantage of the MFCs [1,7].

The power density from a MFC is still quite low in a batch mode operation with synthetic effluent [8].Temperature and pH of the medium, type of electrodes and distance between them, toxicity of the substrate as well as the resistance of the circuit have a significant effect on removal rates and power density of both dual and single chambered MFCs [9,10]. Moreover, the choice of substrate and co-substrates has a profound effect on microbial community profile and output power of MFCs. Even substrates with high organic content derived from biofraction of municipal solid waste under anaerobic conditions can be used for generating methane, hydrogen and electricity under anaerobic conditions [11]. Kook et al. [12] used the liquid fraction of pressed municipal solid waste for generating bioelectricity with an average COD removal of 87%. Dark fermentation effluent is also a favourable substrate for bioelectricity generation using MFCs [3, 13]. Moreover, MFCs can also be used for the selective recovery of metal ions Hg^2+^ or Ag^+^ ions on the cathode [14, 15]. Luo et al. [16] collectively removed Cu^2+^ and Ni^2+^ using MFC coupled with microbial electrochemical cell (MEC).

Earlier, MFCs have been tried for simple substrates but they are now exploited for even toxic and complex substrates such as azo-dyes. Azo-dyes are the most important and largest class of dyes used in commercial applications [17].They are considered as xenobiotics compounds that are very recalcitrant to biodegradation process and most of them are mutagenic and carcinogenic [17, 18]. Hence, their presence in aqueous ecosystem is the cause of serious environmental and health concerns. In the present study, a textile azo-dye acid navy blue r (ANB) used for dyeing wool, nylon or silk was selected for feasibility studies. Unlike aerobic treatment, the azo-dyes get transformed in to corresponding aromatic amines under anaerobic conditions [18].The aromatic amines thus formed are recalcitrant towards further anaerobic degradation but could be mineralized under aerobic conditions resulting in to complete removal [19]. A quick review of the literature also suggested a combined anaerobic-aerobic process for complete solution from dye wastewater [20–22].In most of the cases compounds such as acetate, molasses, or glucose were used as substrate for growth while the toxic azo-dyes were utilized as co-metabolites [23–24]. In this study glucose was chosen as a co-substrate due to its lower toxicity and higher COD which results in higher output power densities [25].

The aim of the work was to check the feasibility of simultaneous electricity generation and complete removal of acid navy blue r dye using a single chambered MFC coupled with an aerobic post treatment process. Efforts have been made to investigate the kinetics and pathway of biodegradation of ANB and the electrochemical behaviour of the cell. The microbial community structures and biofilm growth were studied by using SEM coupled with EDX technique while their quantification was done by the qPCR technique.

## Experimental

### Chemicals

All the chemicals were of analytical grade and procured from Rankem, India. The azo-dye acid navy blue r (ANB) was manufactured by Vipul Dyes, India.

### Reactor Set-up

**Fig 1:**
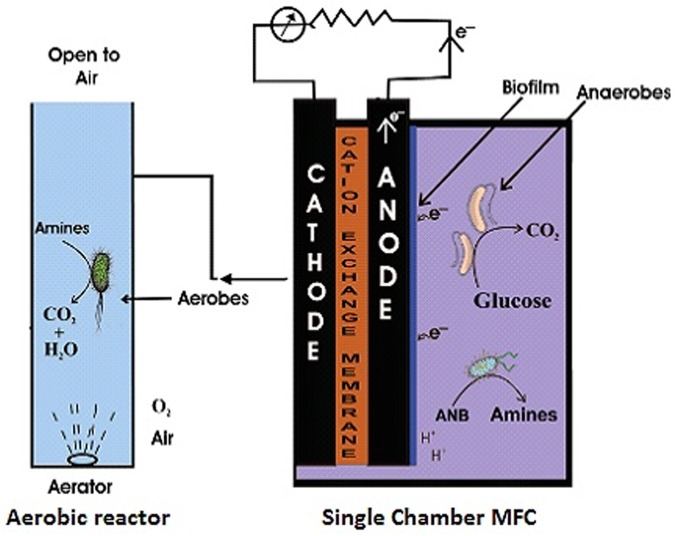
Schematic diagram of the reactor assembly (SMFC coupled with aerobic reactor) used in the present study.

The experimental single chambered MFC set-up used in the present study was made up of a plexi glass chamber of 100 mL volume in which anaerobic conditions were maintained (Fig 1).Two identical graphite rods, cylindrical in shape (area 15.115 cm^2^) were used as electrodes. Graphite anode was inserted in to the plexi glass chamber while graphite cathode was placed on the outer surface of the plexi glass chamber and left open to air. The two electrodes were separated by a proton exchange membrane (PEM Nafion®) placed on the opening of 1.5 cm diameter created on the wall of the plexi glass chamber. In order to complete the external circuit, the electrodes were connected through a copper wire with an external resistance of 470 Ω. The contents of the plexi glass chamber were continuously stirred with the help of a magnetic stirrer. Out of the total volume of SMFC (100 mL), 60 mL was occupied by dye wastewater and 20 mL by anaerobic sludge while 20 mL was left vacant as head space.

The degradation products were further treated in an aerobic reactor consisting of a 100 mL plexi glass chambered open to air. The aerobic mixed liquor was aerated by a domestic air sparger (2 mL/s) in order to maintain the required DO level. The aerobic treatment was given for a period of 48 hr. The aerobic post treatment process was employed to achieve complete degradation of the reduction products of azo-dyes (i.e. aromatic amines).

### Inoculum and basal medium

The anaerobic and aerobic reactors were fed respectively with anaerobic and aerobic (return activated sludge) sludge collected from Okhla sewage treatment plant, New Delhi, India. The mixed liquor suspended solids (MLSS) concentration of anaerobic inoculum was 3 g/L with a very dark coloration however; the aerobic inoculum appears brownish with 5 g/L MLSS concentration. The optical densities for the initial inocula were found to be 1.630 (for anaerobic) and 2.150(aerobic culture). The specific methanogenic activity of the anaerobic culture was 1.8 mM CH_4_/gVSS/d) [26].The composition of the synthetic media used in the SMFC consist of- glucose 1.0 g/L (COD 1050 mg/L per gram of glucose), NH_4_Cl 0.85 g/L, KH_2_PO_4_ 0.136 g/L, K_2_HPO_4_ 0.234 g/L, MgCl_2_.6H_2_O 0.084 g/L, FeCl_3_ 0.05 g/L and Yeast extract 0.34 g/L to fulfill the micronutrient deficiency[27].Four different concentrations of ANB (50, 100, 200 and 400 mg/L)were fed to the SMFC for feasibility studies. The pH, temperature and conductivity remain 7.5–8.0, 27(±2°C and 10.6–18.2 mS/cm throughout the study.

### Analytical methods

The voltage and current across the external resistor was measured with the help of a four channel data recording facility (Kehao KH 200, China). The power density (mW/m^2^) was calculated by dividing the output power with area of electrodes as per the relation given below-

$$PD\;=\frac{V^{2}}{R.A}$$ (1)

Where V is voltage (mV), R is external resistance of the circuit (Ω), A is area of the electrodes (m^2^).

The coulombic efficiency which is the fraction of Coulombs of charge actually transferred with respect to the total coulomb of charge when all the COD is being converted to electricity by assuming the theoretical ratio of 4 mol of electrons per mole of COD, was calculated in accordance with the following relation[20]-

$$CE\;(\%)\;=\frac{M\int_{0}^{t}Idt}{F.b.V_{an}.\Delta COD}$$ (2)

where M is molecular weight of oxygen, I is current, F is Faraday’s constant, V_an_ is the volume of anodic chamber and ΔCOD is the change in COD over time ‘t’.

COD and optical density was measured by visible spectrophotometer (GENESIS 20 Thermo Spectronic, USA) in accordance with the Standard Methods [28]. pH and Conductivity were also monitored using pH and Conductivity meter (Khera Scientific Instruments, India). The treated samples were centrifuged by a micro centrifuge (REMI RM-12C Micro Centrifuge, India) before scanning for the residual concentrations of azo-dye in the wavelength region 200–700 nm in a UV-Visible spectrophotometer (Perkin Elmer Lambda 25, USA). The residual concentrations of ANB were calculated by measuring the absorbance corresponding to N = N at 550 nm(responsible for dark blue coloration) and transforming it to concentration value with the help of a calibration curve (R^2^ = 0.98). Further, the kinetics of decolorization was studied using the rate model and the rate constant values were used to calculate the change in Gibbs free energy of the system (∆G) in order to ensure the spontaneity of the reaction occurring at anode in accordance with the equation given below[1]-

ΔG=−RTlnk (3)

where R, T and k are the universal gas constant (8.314 J/K/mol), temperature at which the experiment was carried out (300 K) and rate constant of the reaction respectively.

The decolorization efficiency and COD removal efficiency were calculated as given below-

$$\%\;COD\;=\frac{{(C}_{0}-C_{t})}{C_{0}}*\;100$$ (4)

$$\%\;DE\;=\frac{{(D}_{0}-D_{t})}{D_{0}}*\;100$$ (5)

where C_0_ and C_t_ are initial and final COD concentrations while D_0_ and D_t_ are initial and final ANB concentrations, respectively.

### Scanning electron microscopy (SEM) and energy dispersive X-ray studies (EDX)

The surface morphology and elemental composition of the anaerobic as well as aerobic sludge were studied by a scanning electron microscope (Jeol JSM-6510) coupled with energy dispersive X-ray. The samples were prepared by SEM-EDX by washing them with sodium cacodylate buffer and fixing them with 2% glutareldehyde overnight [26]. Thereafter, the samples were washed with successive passages of graded ethanol (25, 50, 75, 80, 90 and100%) and dried in a CO_2_ critical point dryer.

### Microbial quantification by the Quantitative real-time polymerase chain reaction (qPCR)

The abundance of the exoelectrogenic *Geobacter* and universal bacterial gene (*Eubacteria*) was estimated by the qPCR technique. Prior to qPCR, the microbial DNA was isolated from initial anaerobic inoculum and anaerobic sludge present in SMFC at the end of the study using the Fast DNA Spin Kit (MP Biomedicals, USA) as per the manufacturer’s instructions. Later, the isolated DNA extract was diluted in the ratio 1:10. Each 10 uL reaction mixture contained:2 μL of template DNA; 0.5 μL of each primer (10 pmol/μL); 2 μL nuclease free water; and 5 μL qPCR reagent (SsoFast EvaGreen Supermix, Biorad, USA). The qPCR was done using the BioRad CFX C1000 (Hercules, CA, USA) system and the following program was loaded-

*Eubacteria*; initial enzyme activation at 95°C for 5 min, denaturation at 95°C for 45 s, annealing at 60.5°C for 45 s and extension step at 72°C for 45 s. The reaction was continued for another 39 cycles. The following sequence of primers was used for *Eubacteria*: Bact338f- ACTCC TACGG GAGGC AG [29] and Bact1046r- CGACARCCATGCANCACCT [30].

*Geobacter*; initial denaturation at 94°C for 4 min, this step is followed by touchdown program consisting of 20 cycles of 94°C for 30s, 65°C for 30s (decreasing by 0.5°C per cycle), and 72°C for 30s. A production program consisting of 15 cycles of 94°C for 30s, 55°C for 30s, and 72°C for 3 min. The following primers were used to target geobacter species: Geo564F- AAGCGTTGTTCGGAWTTA T and Geo840r- GGC ACT GCA GGGGTCAAT A[31].

### Gas chromatography coupled with mass spectroscopy (GC/MS) studies

The products of the biodegradation of ANB in a two stage sequential anaerobic and aerobic process were analyzed in a GC/MS system (Perkin Elmer GC Clarus^®^ 680, USA coupled with Clarus^®^ SQ 8T MS system USA) with a capillary column DB-1 (30mx0.25mmx0.25mm). The samples (4 mL) were extracted with ethyl acetate in the ratio 1:1. The ester fractions were evaporated to dryness and the residue was finally dissolved in 2 ml of methanol for GC/MS analysis. The following temperature program was used for GC/MS analysis- injection temperature 60°C held for 5 min followed by a ramp of 10°C/min to a final temperature of 280°C and held there for 5 min [32].

### Cyclic Voltammetry

Cyclic voltammetry (CV) was used to study the *in situ* electrochemical behaviour of the system using a Cyclic Voltammeter (CHI600D, USA) linked to a data acquisition system. The anode and cathode of the SMFC were taken as working and counter electrodes respectively against an Ag/AgCl reference electrode. The CV was performed at the scan rate of 10 mV/s over a potential ranging from -0.6 V to +0.9 V.

## Results and Discussion

### Bioelectricity generation

**Fig 2:**
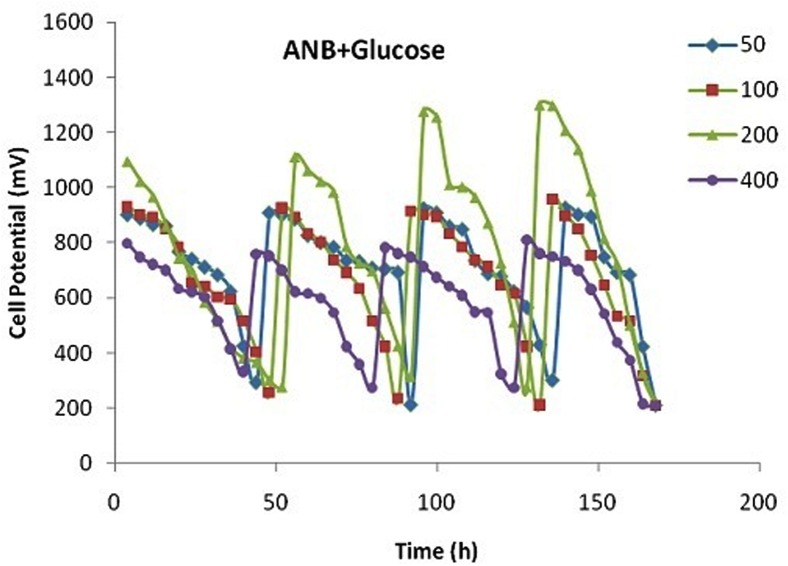
Changes in closed circuit cell potential during the treatment of ANB in a SMFC.

The electricity generation during treatment of ANB in a combined process in presence of glucose was monitored continuously over the test duration in terms of cell potential (mV) and presented in Fig 2. The sludge used was already acclimated for a period of 15 days before the start of the experimental phase. During the acclimation phase the open circuit potential reached to 1350 mV on day 15. Thereafter the circuit was closed with an external load of 470 Ω and the experiment was started. Since the sludge was already acclimated towards ANB, the initial closed circuit cell potential values were quite high.

Four different concentrations of ANB- 50, 100, 200 and 400 ppm were given to the SMFC along with glucose (1 g/L) generating electrical potential in the circuit. The cell potential values shows linear increasing trend with concentration of ANB, attaining a maximum value at 200 ppm and decreases with further rise in ANB concentration. The peak values for 50, 100, 200 and 400 ppm of ANB were 900, 950, 1300, 800 mV respectively. Fresh glucose was added once the potential drops below 200 mV. The peaks shown in Fig 2 represent fresh addition of glucose. The plots are quite similar to the plots shown by Chae et al.[25].However, the output voltage is higher in the present case due to the fact that glucose is most favourable substrate in MFC followed by acetate, propionate and butyrate[33, 34].This also shows that glucose was primarily consumed for generating electricity in the present case.

The output cell potential decreases towards the end of the experiment due to substrate limitations (Fig 2). Thus, a sufficient feast to famine ratio should be maintained for generating bioelectricity in a microbial fuel cell. Moreover, very high concentrations of toxic substrates pose a negative effect on the performance of MFC [2].

### Power density and COD profile

**Fig 3:**
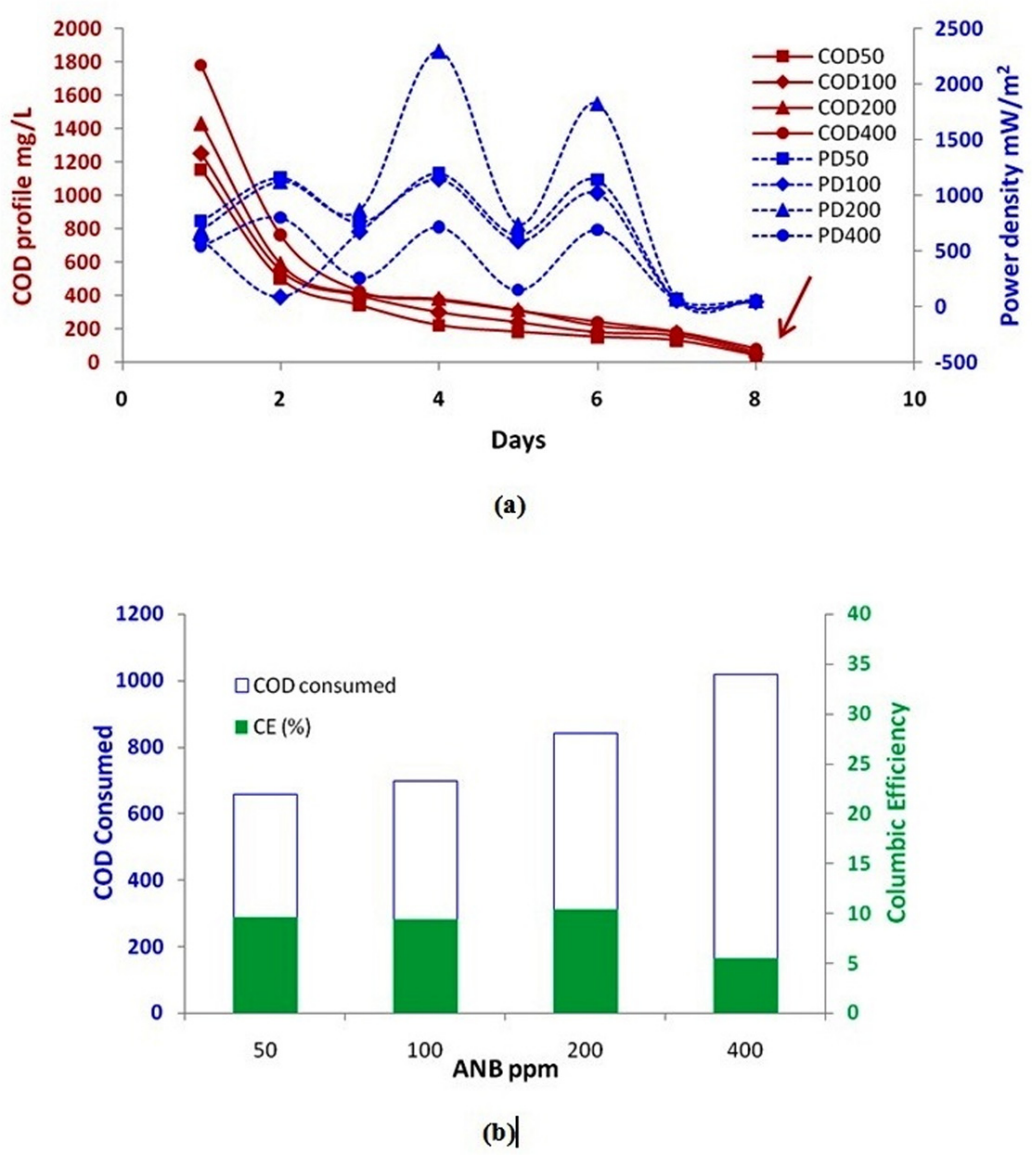
(a) Power density in closed circuit mode along with COD profile during the SMFC treatment of ANB and (b) Columbic efficiency in closed circuit mode along with COD consumed during the treatment of ANB in SMFC coupled aerobic process.

The power density was calculated under a resistance load of 470 Ω and was linked to the amount of COD removed from the MFC, since a part of COD removed is being converted to electricity and the rest is utilized for the growth of biomass (cell division). The power density is a very important parameter for evaluating the performance of MFC for its large scale applications. From Fig 3A, it is clear that power density is inversely proportional to COD profile. The peaks (maxima) in power density plots show fresh addition of glucose while the arrow is pointing towards the COD value attained after aerobic post treatment process. Among all the test concentrations, the power density was highest for 200 ppm of RO16 (2299 mW/m^2^across a resistance of 470 Ω) showing better removal rates at 200 ppm which is consistent with our previous results [2]. However, further rise in dye concentrations beyond 200 ppm led to inhibition of microbial population resulting in lower power densities as mentioned earlier in case of cell potential. When all the COD is being removed, the power density attained a limiting value owing to substrate limitations. Our results shows significant amount of power produced during MFC treatment of an azo-dye in presence of glucose as growth substrate. The acclimated culture gave higher values of power density from day 1. Ringeisen et al. [35] reported a maximum power density of 3000 mW/m^2^ when suspended *S*. *Oneidensis* was fed with10–30mM sodium lactate in anodic medium. On the other hand, Nevin et al. [36]degraded acetate (10 mM) at anode in presence of *Geobacter sulfurreducens* and obtained maximum power density of 1900 mW/m^2^.Dewan et al.[37]reported that power densities could not be increased just by building bigger MFCs since the power density does remain constant when electrode size is increased. They further reported that a capacitor can be used for storing energy from MFC which can later used for powering wireless scientific devices for environmental monitoring. Cusick et al. [38]estimated the cost of electricity produced during treatment of winery and domestic waste water by MFC and reported that the net value of electricity recovered from MFCs could be around $0.026/kg_COD_ for winery wastewater and $0.021/kg_COD_ for domestic wastewater. Thus, the electricity recovered from MFC could be used to reduce the treatment cost.

The coulombic efficiency (CE) of the system was lower as compared to the values reported in literature due to the toxic nature of the azo-dye [20, 25].The highest coulombic efficiency of 10.36%was obtained for 200 ppm of ANB(Fig 3B), which is slightly lower than the value reported by Bakhshian et al.[20] but much higher than the values reported by Zhang et al.[39].The toxic effects are quite significant at higher concentrations of ANB (e.g. 400 ppm) resulting in to lower CE value. Chaudhuri and Lovley [40] observed a CE value of more than 80% in a MFC using *Rhodoferax ferrireducens*. The lower CE values in our case were due to the electron losses attributed either to the presence of electron acceptors (SO_4_^2-^, O_2_ etc) or competing methanogenesis reactions associated with the glucose (fermentable sugar) or both[41].

### COD removal efficiency and dye decolorization

**Fig 4:**
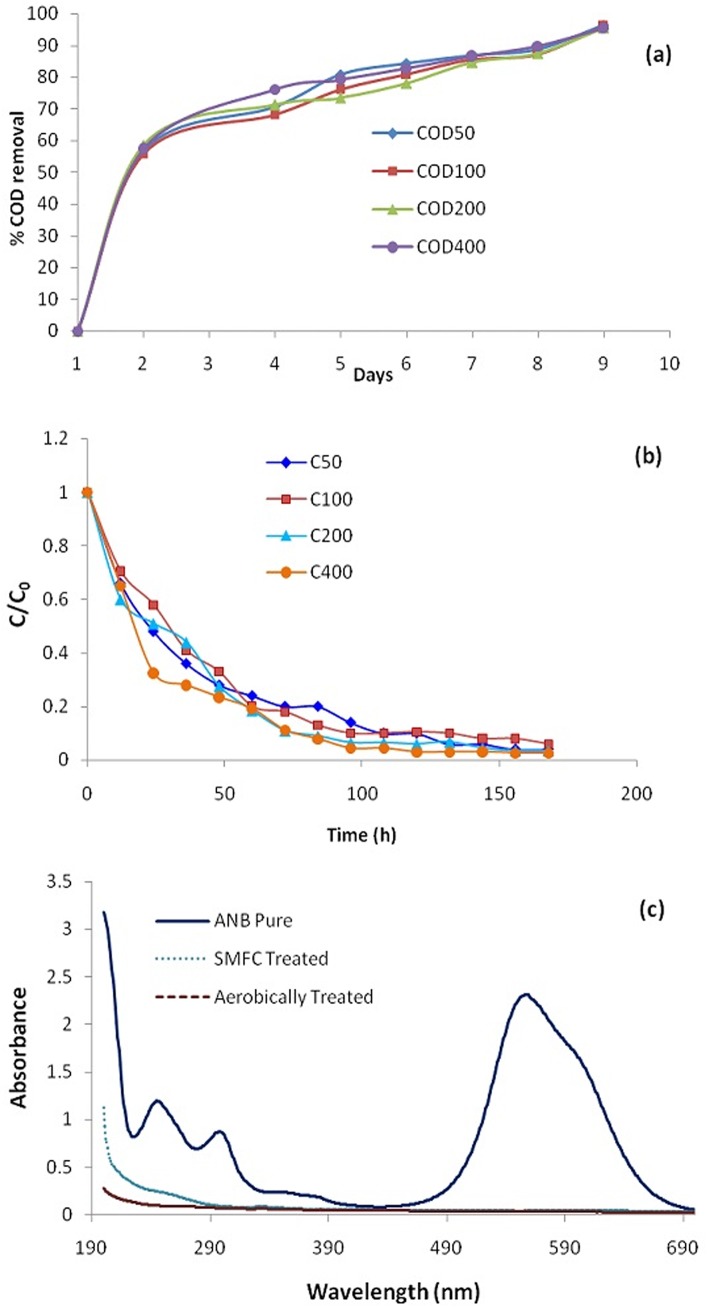
(a) COD removal efficiency, (b) decolorization kinetics and (c) UV spectra of the pure ANB and treated samples.

The per cent COD removal efficiency during ANB degradation in a SMFC was calculated and presented in Fig 4A.The removal efficiency was low initially but as soon as the system got stable the COD removal efficiency was increased and finally stabilized above 80% in SMFC for all the test concentrations. However, in order to achieve removal efficiency of more than 90%, the effluent from the SMFC was fed to aerobic reactor for further treatment. The adopted strategy successfully treated an organic loading rate of 0.254 Kg_COD_/m^3^/d (corresponding to 400 ppm ANB combined along with 1 g/L glucose in the synthetic medium). The COD removal efficiency is directly related to the power density i.e. the higher the removal efficiency, higher will be the output power densities.

Further, the kinetics of decolorization was studied and the rate constant ‘k’ was estimated by plotting C/C_0_ *vs* time (Fig 4B). The decolorization reaction followed pseudo-first order kinetics and the rate constants (k) were found to be 4.4x10^-3^, 4.5x10^-3^, 4.2x10^-3^and 4.4x10^-3^h^-1^ for 50, 100, 200 and 400 ppm of ANB, respectively. Similar behaviour was reported by various researchers [42, 43]. Hosseini Koupaie et al. [44] reported the decolorization of Acid Red 18 to be first order under sequential anaerobic-moving bed biofilm reactor. In contrast, few researchers have used different models for studying the decolorization rate of dyes e.g. Monod model [45].Moreover, the negative values of the thermodynamic parameter ∆G (-13.5, -13.4, -13.6 and -13.6 kJ/mol for 50, 100, 200 and 400 ppm of ANB respectively) estimated from rate constant (k) values show the continuous and spontaneous nature of the reaction occurring in the anodic chamber. The \G is further related to the chemical potential of the system which is defined as the change in free energy with change in composition of the system. In this way, complete decolorization of ANB was achieved in SMFC.

However, the complete decolorization does not always ensure complete degradation since the decolorized products (aromatic amines) get accumulated in to the system under anaerobic conditions. A few authors have reported that long retention time may cause degradation of aromatic amines under anaerobic environments as well [25, 46, 47]. Therefore, in order to ensure speedy and complete degradation (removal) of ANB a different approach has been adopted in the present case by transferring the decolorized effluent to an aerobic reactor (activated sludge process) for further treatment.

The UV spectra of the untreated sample (pure ANB), effluent from the SMFC and the aerobic reactors are presented in Fig 4C. The pure dye shows two peaks at 250 and 290 nm corresponding to benzene and naphthalene rings in the UV region while that at 550 due to the azo linkage in the visible region [48]. Presence of some shoulder peaks in the SMFC effluent represents some organic moiety while absence of any characteristic peak in the aerobically treated samples shows complete degradation of the azo-dye. Further structural identification of the products of biodegradation was done with the help of GC/MS technique.

### Surface morphology and elemental composition

Surface morphology of the microbial sludge collected from SMFC and aerobic reactors was studied with the help of SEM while EDX was used to determine its elemental composition. The results of SEM and EDX analysis are presented in Fig 4.When the system was replenished with fresh glucose, the cell potential and degradation rate increases rapidly due to the sudden change in the microbial activity in the single chambered MFC.

**Fig 5:**
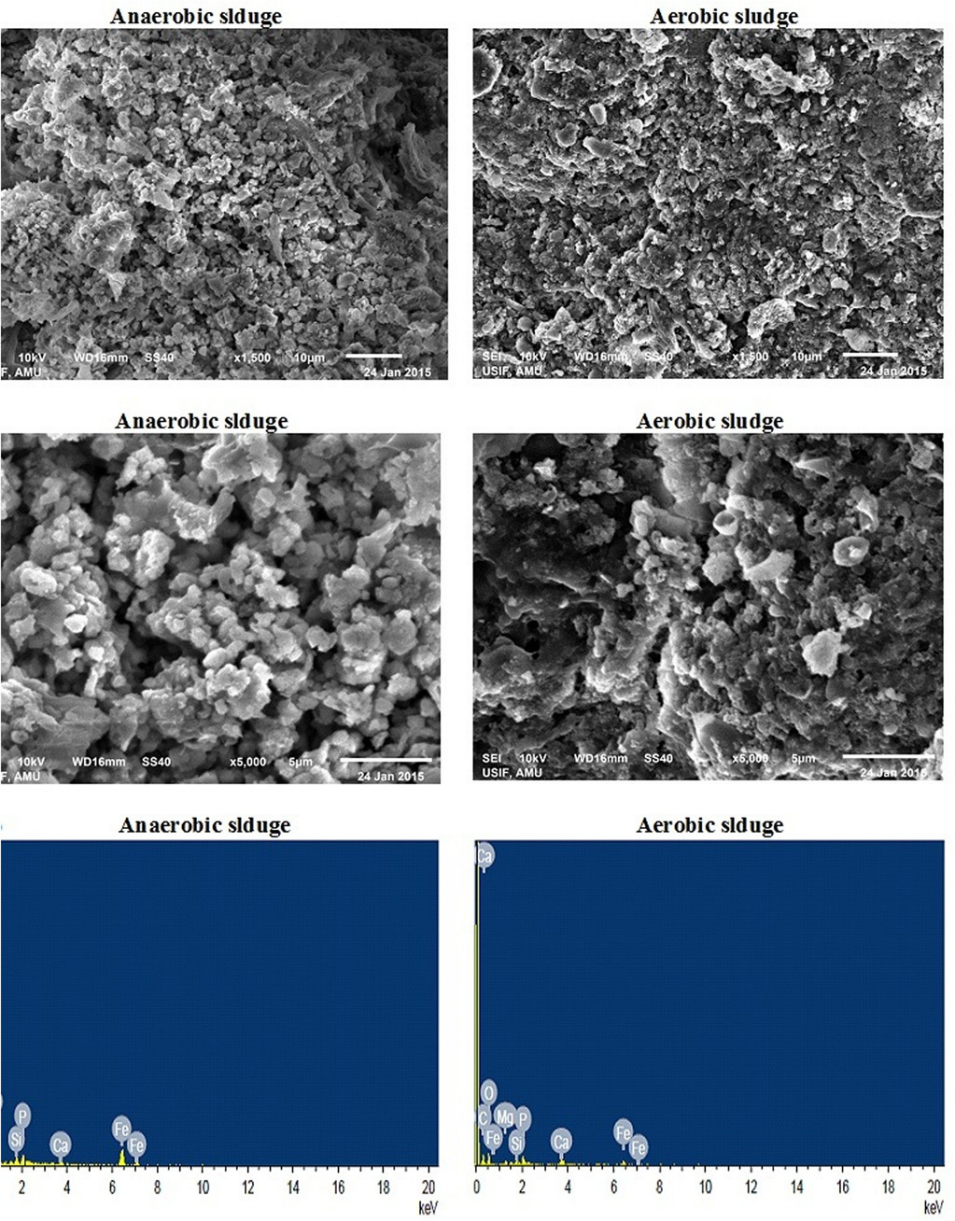
SEM images of the anaerobic and aerobic sludge at magnification of (a) 1500x and (b) 5000x; (c) Elemental composition of the sludge by EDX.

The SEM images of anaerobic and aerobic sludge shows the presence of large number of cocci, diatoms and rod shaped bacteria in the microbial sludge (Fig 5A).However, there is a significant difference in the appearance of sludge taken from SMFC and aerobic reactor due to the distinct nature of microorganisms and different set of operating conditions (e.g. different composition of inlet feed). At a magnification of 5000x, some sort of channelling appeared in the SEM microstructures shown in Fig 5B which allows better mass transfer resulting in to better degradation rates which might be a strong reason for higher output voltage and power densities in the present case. The EDX spectra show the presence of various elements such as C, O, Fe, Mg, Ca, Si, K etc (Fig 5B). These elements are present in the extra cellular polymeric substance secreted by microorganisms. However, few metals shown in EDX spectra were originated from the synthetic media used in the present study.

**Fig 6:**
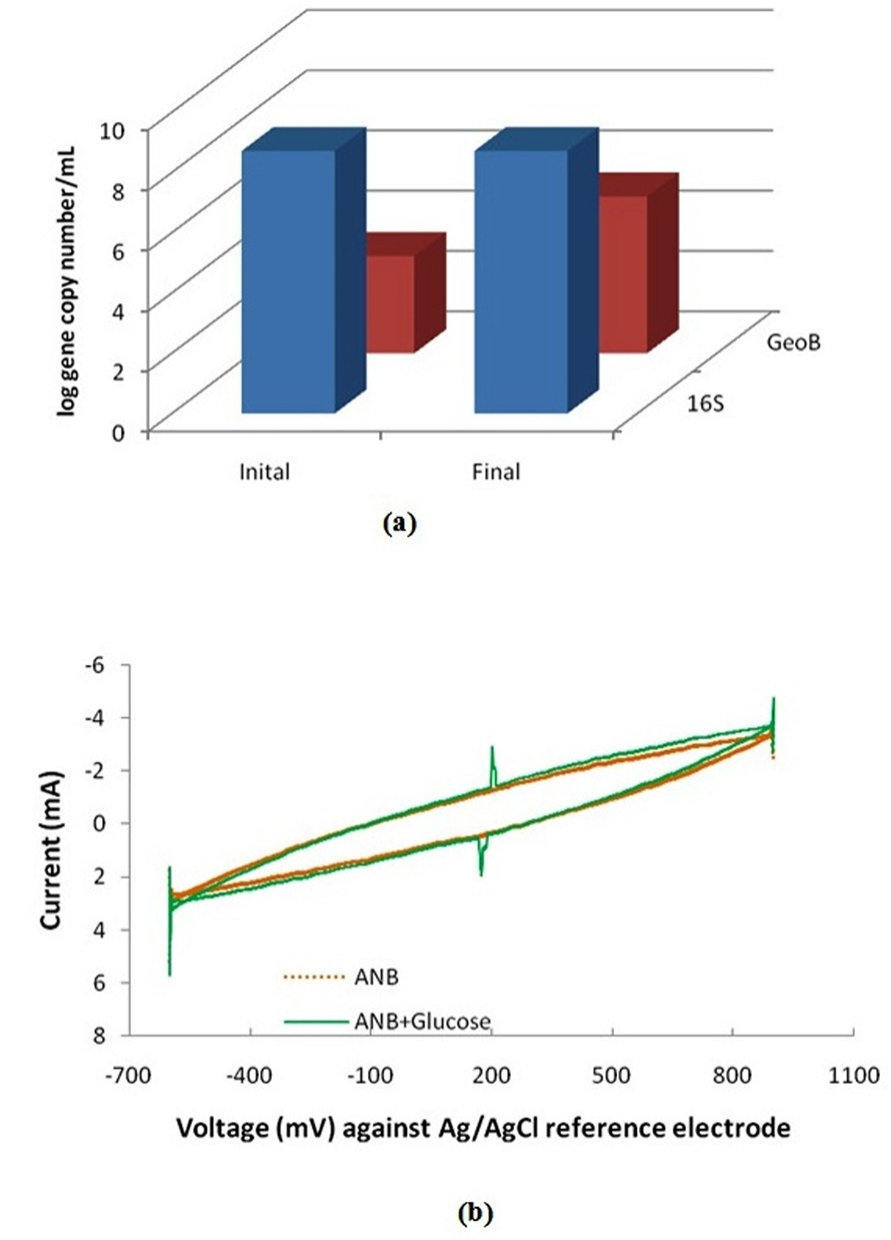
(a) Population of the universal bacterial gene and electrochemically active *Geobacter* species in the anaerobic sludge treating ANB, (b)Electrochemical characterization of the fuel cell (cyclic voltammograms).

Further, the absolute abundance of the universal bacterial gene and the exoelectrogenic *Geobacter* was estimated using the qPCR technique and presented in the Fig 6A in terms of log gene copy number/mL. The qPCR technique is a powerful technique for quantification of the microbial communities in a real time process and hence finds use in numerous fields. The technique was applied to the initial anaerobic sludge inoculated in to the SMFC and the sludge obtained at the end of the experiment so as to quantify the growth or inhibition of microbial communities. Although, the sludge may contains varied classes of bacteria, only the electrochemically active *Geobacter* mainly responsible for electricity production was estimated along with the universal bacterial communities so as to check the relative abundance of *Geobacter* among the total bacterial population. From Fig 6A it is clear that the population of the *Geobacter* has been greatly increased during the course of the study by supplying electrode as electron acceptors. The *Geobacter* plays a vital role in bioelectricity generation by efficiently transferring the electrons to the positive electrode (anode).

### Cyclic voltammetry

The electrochemical behaviour of the fuel cell was studied by the *in situ* cyclic voltammetry technique(Fig 6B).The voltammetry was performed for the bulk anolyte solution (containing ANB and glucose)as well as for ANB alone without any replacement by taking the anode as working electrode against cathode as counter electrode and Ag/AgCl as reference electrode. A perfect redox loop was obtained in both the voltammogram shown in Fig 6B confirmed the critical role played by anaerobic microorganism in transferring the electrons to the anode. Absence of any peak corresponding to anodic or cathodic currents in case of anolyte solution containing ANB only (without co-substrate glucose) shows that it was not primarily oxidized at anode. However, peaks observed in case of bulk anolyte solution (containing ANB and glucose both) shows that glucose was preferentially consumed as substrate for growth and current production while ANB was utilized as a co-metabolite. Moreover, the smaller values of anodic and cathodic peak currents in Fig 6B (as shown by the small sizes of the peaks) are due to the absence of any redox mediator used in the present study[25].Thus, mediators have significant effect on anodic or cathodic current in bioelectrochemical systems. In addition to chemical mediators, certain microorganism can also support the electron transfer process [4, 49].

### Investigation of the pathway of biodegradation

Investigation of the degradation pathway is very essential in order to determine the structure and nature of products formed and to ensure complete removal of azo-dye. The formation of the products depends upon the diversity of microorganisms and the experimental conditions. Under anaerobic conditions, the azo-bond (N = N) of acid navy blue r is more susceptible to microbial attack resulting in to disappearance of colour along with the formation of aromatic amines in the SMFC which is also confirmed by the UV spectra of the treated samples [2, 50, 51].The aromatic amines are also reported during photooxidation of azo-dyes under UV irradiation [32].These aromatic amines got accumulated under anaerobic conditions in to the SMFC and remain unaffected. Therefore, in order to achieve complete and timely degradation of acid navy blue r, the contents of the SMFC were further treated in an activated sludge process in order to cleave the aromatic rings also. Svobodova et al. [52] reported the opening of ring structure during degradation of the azo-dye by *I*. *lacteus* owing to MnP activity present in *I*. *lacteus*.

In the present case, the azo-dye ANB was transformed in to 1-naphthaleinamine (m/z 143), Broener’s acid (m/z 223) and aniline (m/z 93) in the SMFC. These amines were further degraded in the aerobic post treatment process yielding two different products at m/z 166 and 223. The two products were identified as phthalic acid and its derivative diethyl phthalate respectively using the NIST Library. The MS spectra of the products of biodegradation of ANB in the present study are shown in the S1 Fig.

**Fig 7:**
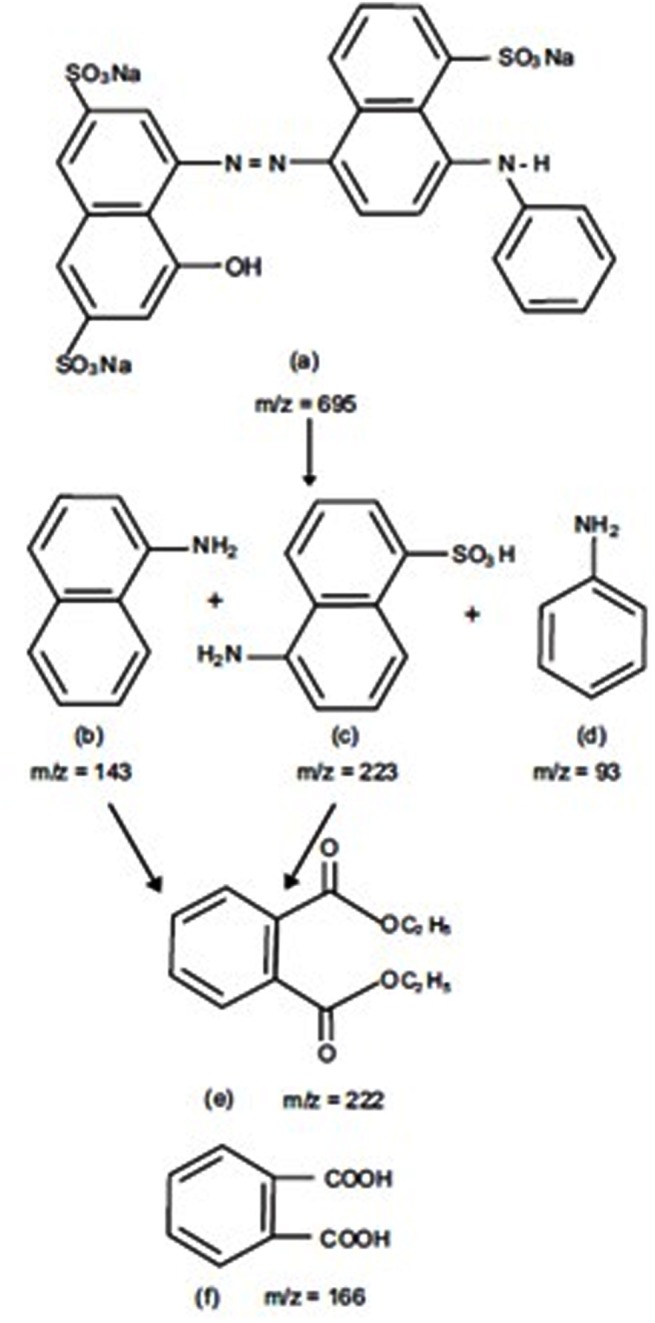
Probable degradation pathway of acid navy blue r based on GC/MS data (a- Acid navy blue r; b- 1-Naphthaleinamine; c- Broener’s acid; d- Aniline; e- Diethyl phthalate and f-Phthalic acid).

Based on the results of GC/MS studies, a biodegradation pathway of ANB under combined anaerobic-aerobic conditions has been proposed in Fig 7. Thus complete removal of azo-dye requires a combination of processes involving both the anaerobic and aerobic conditions.

## Conclusions

Bioelectricity generation can be made possible during the biodegradation of ANB in a single chambered MFC in presence of a co-substrate. This is the first study of ANB in a combined process consisting of SMFC followed by an activated sludge system. The maximum cell potential and coulombic efficiency were 1300 mV and 10.36% for 200 ppm of ANB. The power density is directly related to the COD removal efficiency and peaked at 2236 mW/m^2^. The kinetic and thermodynamic parameters favour better decolorization of the azo dye in the SMFC. The adopted strategy involving two stage processes was proven successful in this regard.

The effluent from the SMFC was further treated in an aerobic post treatment process for complete removal of the azo-dye and the products were identified by GC/MS techniques. The initial microbial attack transformed the azo-dye in to corresponding amines which were further degraded to form phthalic acid and its derivatives as end products. Thus, SMFC technology is a feasible alternative for simultaneous electricity generation and dye degradation. The presence of electrode as terminal electron acceptors favours high degradation rates; however, the presence of oxygen must be avoided in the anodic chamber to evade electron losses. In future, the proposed strategy can be applied as low cost waste water treatment system.

## Supporting Information

S1 Fig MS spectra of the degradation products (a) 1-naphthaleinamine m/e 143; (b) Broener’s acid m/e 223; (c) Aniline m/e 93; (d)- Diethyl Phthalate m/e 222 and (e) Phthalic acid m/e 166. (TIF)

## References

[1] Logan B, Hamelers B, Rozendal R, Schroder U, Keller J, Freguia S, et al (2006a) Microbial Fuel Cells: Methodology and Technology. Envi Sci Technol 40: 5181–92. 10.1021/es0605016 16999087

[2] Khan MZ, Singh S, Sreekrishnan TR, Ahammad SZ (2015) Studies on biodegradation of two different azo dyes in bioelectrochemical systems. New J Chem (in press) 10.1039/C5NJ00541H

[3] Rivera I, Buitron G, Bakonyi P, Nemestothy N, Belafi-Bako K (2015) Hydrogen production in a microbial electrolysis cell fed with a dark fermentation effluent. J Appl Electrochem (in press) 10.1007/s10800-015-0864-6

[4] Rabaey K, Boon N, Siciliano SD, Verhaege M, Verstraete W (2004) Biofuel cells select for microbial consortia that self-mediate electron transfer. Appl Environ Microbiol 70: 5373–5382. 15345423 10.1128/AEM.70.9.5373-5382.2004 PMC520914

[5] Bond DR, Lovley DR (2003) Electricity production by *Geobacter sulfurreducens* attached to electrodes. Appl Environ Microbiol 69: 1548–1555. 12620842 10.1128/AEM.69.3.1548-1555.2003 PMC150094

[6] Gorby YA, Yanina S, McLean JS, Rosso KM, Moyles D, Dohnalkova A, et al (2006). Electrically conductive bacterial nanowires produced by *Shewanella oneidensis* strain MR-1 and other microorganisms. PNAS 103: 11358–11363. 16849424 10.1073/pnas.0604517103 PMC1544091

[7] Zhang F, Cheng S, Pant D, Bogaert GV, Logan BE (2009) Power generation using an activated carbon and metal mesh cathode in a microbial fuel cell. Electrochem Commun 11: 2177–2179.

[8] Logan BE (2008) Microbial Fuel Cells, John Wiley & Sons, Inc., Hoboken, New Jersey, USA.

[9] Wu C, Liu XW, Li WW, Sheng GP, Zang GL, Cheng, et al (2012) A white-rot fungus is used as a biocathode to improve electricity production of a microbial fuel cell. Appl Energy 98: 594–596.

[10] Sevda S, Benetton XD, Vanbroekhoven K, Sreekrishnan TR, Pant D (2013) Characterization and comparison of the performance of two different separator types in air–cathode microbial fuel cell treating synthetic wastewater Chem. Eng. J. 228: 1–11.

[11] Rozsenberszki T, Kook L, Hutvagner D, Nemestothy N, Belafi-Bako K, Bakonyi P, et al (2015) Comparison of Anaerobic Degradation Processes for Bioenergy Generation from Liquid Fraction of Pressed Solid Waste. Waste Biomass Valorization 6: 465–473.

[12] Kook L, Rozsenberszki T, Nemestothy N, Belafi-Bako K, Bakonyi P (2015) Bioelectrochemical treatment of municipal waste liquor in microbial fuel cells for energy valorization. J. Clean Prod (in press) 10.1016/j.jclepro.2015.06.116

[13] Elmekawy A, Srikanth S, Vanbroekhoven K, De Wever H, Pant D (2014)Bioelectro-catalytic valorization of dark fermentation effluents by acetate oxidizing bacteria in bioelectrochemical system (BES). J Power Sour 262: 183–191.

[14] Wang ZJ, Lim B, Choi C (2011) Removal of Hg^2+^ as an electron acceptor coupled with power generation using a microbial fuel cell. Bioresour. Technol. 102: 6304–6307. 10.1016/j.biortech.2011.02.027 21377357

[15] Tao HC, Gao ZY, Ding H, Xu N, Wu WM (2012) Removal of heavy metals from fly ash leachate using combined bioelectrochemical systems and electrolysis. Bioresour. Technol. 111: 92–97. 10.1016/j.biortech.2012.02.029 24269969

[16] Luo H, Qin B, Liu G, Zhang, Tang Y, Hou Y (2015) Selective recovery of Cu^2+^ and Ni^2+^ from wastewater using bioelectrochemical system. Front. Environ. Sci. Eng. 9: 522–527.

[17] Zollinger H (1991) Color Chemistry: Syntheses, Properties and Applications of Organic Dyes and Pigments, second ed. VHC Publishers, New York.

[18] Pandey A, Singh P, Iyengar L (2007) Bacterial decolorization and degradation of azo dyes. Int Biodeter Biodegr 59: 73–84.

[19] Wang J, Liu GF, Lu H, Jin RF, Zhou JT, Lei TM (2012) Biodegradation of Acid Orange 7 and its auto-oxidative decolorization product in membrane-aerated biofilm reactor. Int Biodeter Biodegr 67: 73–77.

[20] Bhakshian S, Kariminia HR, Roshandel R. (2011) Bioelectricity generation enhancement in a dual chamber microbial fuel cell under cathodic enzyme catalyzed dye decolorization. Bioresour Technol 102: 6761–6765. 10.1016/j.biortech.2011.03.060 21511458

[21] Liu H, Cheng SA, Logan BE (2005) Production of electricity from acetate or butyrate using a single-chamber microbial fuel cell. Environ Sci Technol 39: 658–662. 15707069 10.1021/es048927c

[22] Logan BE, Regan JM (2006b) Electricity-producing bacterial communities in microbial fuel cells. Trends Microbiol 14: 512–518. 17049240 10.1016/j.tim.2006.10.003

[23] Logan BE, Murano C, Scott K, Gray ND, Head IM (2005) Electricity generation from cysteine in a microbial fuel cell. Water Res 39: 942–952. 15743641 10.1016/j.watres.2004.11.019

[24] Lovley DR (2006) Bug juice: harvesting electricity with microorganisms. Nat Rev Microbiol 4: 497–508. 16778836 10.1038/nrmicro1442

[25] Chae KJ, Choi MJ, Lee JW, Kim KY, Kim IS (2009) Effect of different substrates on the performance, bacterial diversity, and bacterial viability in microbial fuel cells. Bioresour. Technol. 100: 3518–3525. 10.1016/j.biortech.2009.02.065 19345574

[26] Khan MZ, Singh S, Sreekrishnan TR, Ahammad SZ (2014). Feasibility study on anaerobic biodegradation of azo dye reactive orange 16. RSC Adv 4: 46851.

[27] Speece RE (1996) Anaerobic biotechnology for industrial wastewaters, Archae Press, Nashville, TN, USA, pp. 1–320.

[28] APHA (2002) Standard Methods for the Examination of Water and Wastewater. 20th edition, American Public Health Association, Washington, D.C. USA.

[29] Yu Y, Lee C, Kim J, Hwang S (2005) Group specific primer and probe sets to detect methanogenic communities using quantitative real-time polymerase chain reaction. Biotechnol Bioeng. 89: 670–679. 15696537 10.1002/bit.20347

[30] Kondo R, Nedwell DB, Purdy KJ, Silva SQ (2004) Detection and enumeration of sulphate-reducing bacteria in estuarine sediments by competitive PCR. Geomicrobiol J., 21: 45–157.

[31] Cummings DE, Snoeyenbos West OL, Newby DT, Niggemyer AM, Lovley DR, et al (2003) Diversity of *Geobacteraceae* species inhabiting metal-polluted freshwater lake sediments ascertained by 16S rDNA analyses. Microbial Ecology 46: 257–269. 14708750 10.1007/s00248-005-8002-3

[32] Bilgi S, Demir C (2005) Identification of photooxidation degradation products of C.I. Reactive Orange 16 dye by gas chromatography–mass spectrometry. Dyes and Pigments 66: 69–76.

[33] Jung S, Regan JM (2007) Comparison of anode bacterial communities and performance in microbial fuel cells with different electron donors. Appl Microbiol Biotechnol 77: 393–402. 17786426 10.1007/s00253-007-1162-y

[34] Liu H, Cheng SA, Logan BE (2005) Production of electricity from acetate or butyrate using a single-chamber microbial fuel cell. Environ Sci Technol 39: 658–662. 15707069 10.1021/es048927c

[35] Ringeisen BR, Ray R, Little B (2007) A miniature microbial fuel cell operating with an aerobic anode chamber. J Power Sources 165: 591–597.

[36] Nevin KP, Richter H, Covalla SF, Johnson JP, Woodard TL, Orloff AL, et al (2008).Power output and coulombic efficiencies from biofilms of *Geobacter sulfurreducens* comparable to mixed community microbial fuel cells. Environ Microbiol 10: 2505–2514. 10.1111/j.1462-2920.2008.01675.x 18564184

[37] Dewan A, Donovan C, Heo D, Beyenal H (2010) Evaluating the performance of microbial fuel cells powering electronic devices. J Power Sour 195: 90–96.

[38] Cusick RD, Kiely PD, Logan BE (2010) A monetary comparison of energy recovered from microbial fuel cells and microbial electrolysis cells fed winery or domestic wastewaters. International Journal of hydrogen energy 35: 8855–8861.

[39] Zhang B, Zhao H, Zhou S, Shi C, Wang C, Ni J (2009) A novel UASB-MFC-BAF integrated system for high strength molasses wastewater treatment and bioelectricity generation. Bioresour Technol 100: 5687–5693. 10.1016/j.biortech.2009.06.045 19604688

[40] Chaudhuri SK, Lovley DR (2003) Electricity generation by direct oxidation of glucose in mediator less microbial fuel cells. Nat Biotechnol 21: 1229–1232. 12960964 10.1038/nbt867

[41] Pant D, Bogaert GV, Diels L, Vanbroekhoven K (2010) A review of the substrates used in microbial fuel cells (MFCs) for sustainable energy production. Bioresour. Technol. 101: 1533–1543. 10.1016/j.biortech.2009.10.017 19892549

[42] Yu L, Li WW, Lam MHW, Yu HQ (2011) Adsorption and decolorization kinetics of methyl orange by anaerobic sludge. Appl Microbiol Biotechnol 90: 1119–1127. 10.1007/s00253-011-3109-6 21279343

[43] Murali V, Ong SA, Ho LN, Wong YS (2013) Evaluation of integrated anaerobic–aerobic biofilm reactor for degradation of azo dye methyl orange. Bioresour Technol 143: 104–111. 10.1016/j.biortech.2013.05.122 23792659

[44] Hosseini Koupaie E, Alavi Moghaddam MR, Hashemi SH (2012) Investigation of decolorization kinetics and biodegradation of azo dye Acid Red 18 using sequential process of anaerobic sequencing batch reactor/moving bed sequencing batch biofilm reactor. Int Biodeterior Biodegrad 71: 43–49.

[45] Hsueh CC, Chen BY (2007) Comparative study on reaction selectivity of azo dye decolorization by *Pseudomonas luteola* . J Hazard Mater 141: 842–849. 16949740 10.1016/j.jhazmat.2006.07.056

[46] Razo-Flores E, Luijten M, Donlon BA, Lettinga G, Field JA (1997) Complete biodegradation of the azo dye azodisalicylate under anaerobic conditions. Environ Sci Technol 31: 2098–2103. 46858.

[47] Isik M, Sponza DT (2007) Fate and toxicity of azo dye metabolites under batch long-term anaerobic incubations. Enzyme Microb Technol 40: 934–939.

[48] Mitrovic J, Radovic M, Bojic D, Andelkovic T, Purenovic M, Bojic A (2012) Decolorization of textile azo dye reactive orange 16 with UV/H2O2 process.J Serb Chem Soc 77: 465–481.

[49] Rabaey K, Boon N, Hofte M, Verstraete W (2005) Microbial phenazine production enhances electron transfer in biofuel cells. Environ Sci Technol 39: 3401–3408. 15926596 10.1021/es048563o

[50] Georgiou D, Metallinou C, Aivasidis A, Voudrias A, Gimouhopoulos K (2004) Decolorization of azo-reactive dyes and cotton-textile wastewater using anaerobic digestion and acetate-consuming bacteria. Biochem Eng J 19: 75–79.

[51] Mendez-Paz D, Omil F, Lema JM (2005) Anaerobic treatment of azo dye Acid Orange 7 under batch condition. Enzyme Microb Technol 36: 264–272. 10.1016/j.watres.2004.11.022 15743621

[52] Svobodova K, Senholdt M, Novotny C, Rehorek A(2007) Mechanism of Reactive Orange 16 degradation with the white rot fungus *Irpex lacteus* . Process Biochem 42: 1279–1284.

