# Supplementary figures and images for: Bioelectricity Generation and Bioremediation of an Azo-Dye in a Microbial Fuel Cell Coupled Activated Sludge Process
Source: PLoS One. 2015 Oct 23;10(10):e0138448. doi: 10.1371/journal.pone.0138448 (PMC4619775; doi:10.1371/journal.pone.0138448)

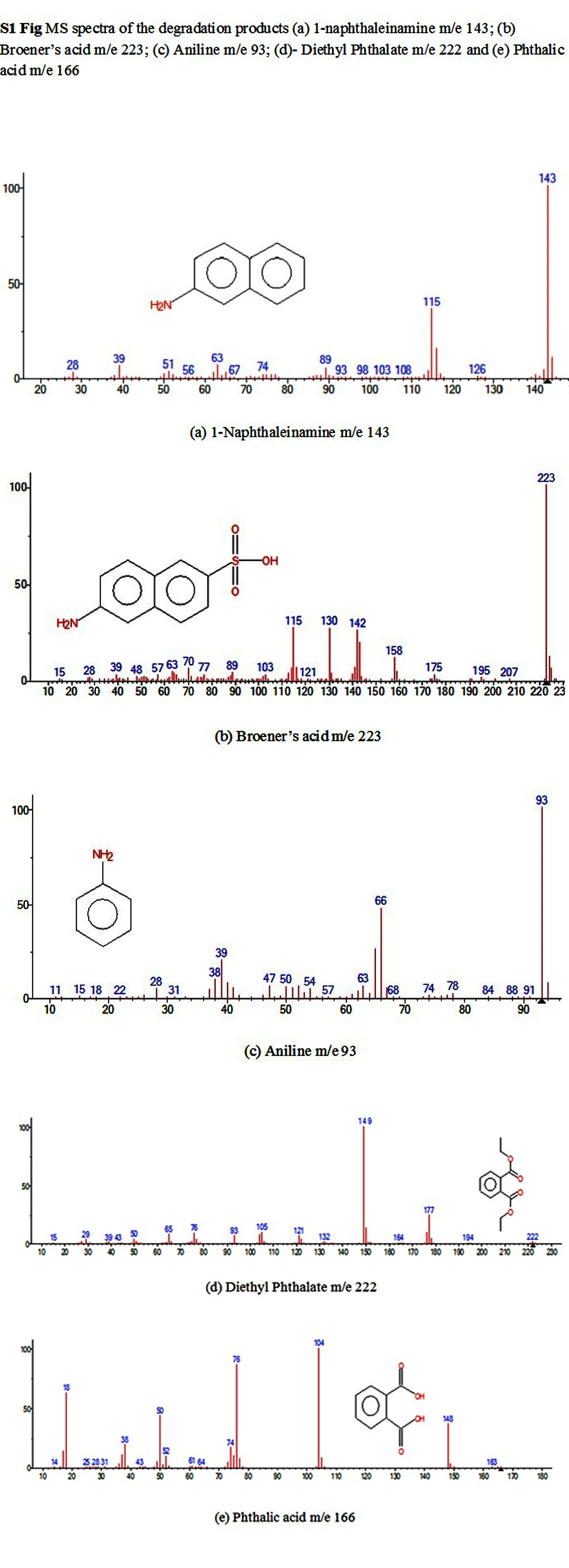

Supplement: S1 Fig — (TIF) [file pone.0138448.s001.tif]
